# Intravital Multiphoton Examination of Implant-Associated *Staphylococcus aureus* Biofilm Infection

**DOI:** 10.3389/fcimb.2020.574092

**Published:** 2020-10-15

**Authors:** Casey M. Gries, Zuivanna Rivas, Justin Chen, David D. Lo

**Affiliations:** Division of Biomedical Sciences, School of Medicine, University of California, Riverside, Riverside, CA, United States

**Keywords:** multiphoton microscopy, medical device infection, *Staphylococcus aureus*, biofilm, innate immunity

## Abstract

Bacterial infections associated with implanted medical devices represents a healthcare crisis due to their persistence, antibiotic tolerance, and immune avoidance. Indwelling devices are rapidly coated with host plasma and extracellular matrix proteins which can then be exploited by bacterial pathogens for adherence and subsequent biofilm development. Our understanding of the host-pathogen interface that determines the fate of biofilm-mediated infections is limited to the experimental models employed by laboratories studying these organisms. Current *in vivo* models of biofilm-mediated infection, while certainly useful, are typically limited to end-point analyses of bacterial burden enumeration, immune cell profiling, and cytokine/chemokine analysis. Thus, with these models, the complex, real-time assessment of biofilm development and innate immune cell activity remains imperceptible. Here, we describe a novel murine biofilm infection model employing time-lapse intravital multiphoton microscopy which permits concurrent and real-time visualization of *Staphylococcus aureus* biofilm formation and immune cell activity. Using cell tracking, we found that *S. aureus* biofilms impede neutrophil chemotaxis, redirecting their migration patterns to prevent biofilm invasion. This approach is the first to directly examine device-associated biofilm development and host-pathogen interactions and will serve to both further our understanding of infection development and help reveal the effects of future antibiofilm treatment strategies.

## Introduction

Bacterial biofilm infections remain a significant healthcare problem worldwide. Infection risk is substantially increased in the presence of an implanted medical device, such as an orthopedic prosthesis, electronic cardiac device, artificial heart valve, or indwelling catheter (Costerton et al., 2005; Tande and Patel, 2014; Arciola et al., 2018). Over 25% of nosocomial infections are associated with an implanted medical device (Magill et al., 2014), and the incidence of infected hip and knee arthroplasty rates are expected to rise (Kurtz et al., 2007; Tande and Patel, 2014). The current standard-of-care involves surgical debridement and, if necessary, a two-step removal of the infected hardware including placement of a temporary spacer, 4–8 weeks of parenteral antimicrobial therapy, followed by insertion of a new device. This long and debilitating process is associated with significant patient morbidity and financial burden, often exceeding $90,000 per infection (Kurtz et al., 2012). In addition, prior device-associated infection increases patient risk for infection relapse. Thus, an urgent need exists for novel approaches to prevent device-associated infection and/or facilitate biofilm eradication.

The leading causes of bacterial device-associated infection are *Staphylococcus* sp, *Enterococcus faecalis*, and *Pseudomonas aeruginosa* (Arciola et al., 2018). These pathogens vary widely in their biochemistry, antimicrobial sensitivities, and virulence mechanisms employed to cause disease. Moreover, device-associated infections often pose difficulties in diagnosis using classic culture techniques (Fernandez-Sampedro et al., 2017), however, new molecular methodologies have shown promise in permitting accurate pathogen identification (Costerton et al., 2011). While staphylococci are the leading cause of prosthetic joint infections (Arciola et al., 2018), the causative species of other implant-associated infections largely depend on the implant site and type, patient co-morbidities, geographical location, and time since surgery (Aggarwal et al., 2014).

Medical implants are rapidly coated with host extracellular matrix molecules that help modulate a foreign body reaction, but also represent a surface target for bacterial attachment. As opposed to the planktonic lifestyle, biofilms are adherent communities of bacteria, often encased within a self-produced matrix of DNA, proteins, and/or polysaccharides. The biofilm mode of growth is regulated by a complex network of genetic factors responding to various environmental cues, including available metabolites, host molecules, and quorum sensing (Stewart and Franklin, 2008; Arciola et al., 2012). Moreover, biofilms are notoriously recalcitrant to antibiotic therapy due to metabolic heterogeneity and are well-described for their ability to resist innate immune defenses, including leukocyte invasion and phagocytosis (Thurlow et al., 2011; Mulcahy et al., 2014; Gries and Kielian, 2017). Three-dimensional biofilm structure not only poses a physical barrier to host immune cell infiltration and phagocytosis (Gries et al., 2020), but biofilm products can actively skew immune responses to enable infection persistence (Benoit et al., 2008; Scherr et al., 2014; Gries et al., 2016).

Many *in vitro* and *in vivo* models to have been developed to study biofilm development and host immune responses (Lebeaux et al., 2013). *In vitro* methods often comprise of static or sheer flow models mimicking infections associated with relatively stationary (e.g., internal fixation, peripheral catheters, etc.) or dynamic (e.g., joint arthroplasty, cardiac valve, vascular catheter, etc.) sites, respectively. These models can also include the addition of host factors or immune cells to assess anti-biofilm activity and/or bacterial responses. *In vivo* methods examining biofilm-mediated infections often involve rodent or rabbit models of device-associated infection. These models are largely limited to *ex vivo* analyses; requiring sacrifice of the animal for end-point quantification, including bacteria burdens, tissue histology, flow cytometry, and/or quantifying cytokine/chemokine production. More recently, continuous monitoring of biofilm infection has been demonstrated with bioluminescent bacterial strains and fluorescent reporter animals using whole-animal *in vivo* imaging systems (Thurlow et al., 2011; Wang et al., 2017; Gutierrez Jauregui et al., 2019). These models are advantageous as they do not require sacrifice of the animal to glean useful data, however they are limited by camera sensitivity and associating bioluminescent image data with established bacterial burden standard curves. In addition, these small-animal imaging systems do not permit cellular-level resolution and therefore rely on large number of congregating bioluminescent or fluorescent cells to emit a detectable signal.

To assess the cellular activities and interactions occurring during biofilm infection, several studies have employed confocal or epifluorescent microscopy (Forestier et al., 2017; Abdul Hamid et al., 2020). Unfortunately, these experiments are restricted to a limited depth penetration and single short-wavelength excitatory light that rapidly damages animal tissues. Unlike confocal and epifluorescent microscopy, multiphoton microscopy (MPM) utilizes simultaneous absorption of two or more long-wavelength photons to produce a single, short-wavelength excitatory stimulus. Longer wavelengths of light enable greater tissue penetration without the damaging effects of confocal/epifluorescent light sources (Denk et al., 1990), thereby permitting time-lapse imaging within living tissues. Furthermore, multiple detectors and spectral imaging confer the ability for spatiotemporal multiplexing and second-harmonic generation. MPM technology has rapidly evolved to include high-speed laser scanning and optical sectioning with up to 1 mm depth penetration, allowing 3-D reconstruction of tissue.

While MPM has proven useful in examining bacterial infection in living animals (Hickman et al., 2009; Abtin et al., 2014; Stolp and Melican, 2016), to-date MPM has not been employed to examine bacterial biofilm-mediated infections. Here we report on the use of MPM to simultaneously assess *Staphylococcus aureus* biofilm development and innate immune cell activity. At the time of preparation, this is the first report to examine *S. aureus* biofilm and associated innate immune response using intravital MPM. Furthermore, previous studies have shown a paucity of neutrophil influx at the site of infection (Thurlow et al., 2011; Hanke and Kielian, 2012), despite their enhanced ability to invade *S. aureus* biofilm *in vitro* compared to macrophages (Gunther et al., 2009; Scherr et al., 2013). We hypothesize that *S. aureus* biofilms modulate neutrophil behavior to promote infection persistence. To test this, we utilized cell tracking during time-lapse MPM to monitor neutrophil migration behavior and determined that neutrophils associated with *S. aureus* biofilm infection migrate randomly, indicating that they are redirected to avoid interaction, permitting biofilm persistence.

## Methods

### Bacterial Strains and Culture Conditions

The wild-type *S. aureus* strain used in this study was LAC-13C, a USA300 MRSA skin and soft tissue infection isolate cured of plasmid p03 (Fey et al., 2013), widely used in biofilm infection studies with comparable findings to other strains (Vidlak and Kielian, 2016). The plasmid pCM29 was used as a constitutive source of superfolder-GFP (sGFP) expression (Pang et al., 2010). For infection studies, bacteria were prepared by inoculating freshy isolated colonies into 25 mL Brain-Heart Infusion broth (BHI; Oxoid, UK) containing 10 μg ml^−21^ chloramphenicol and cultured for 16 h at 37°C, 250 RPM. Cells were then washed twice with PBS and diluted to 5 × 10^5^ CFU mL^−1^ prior to infection.

### Animals

The wild-type C57Bl/6 and CX3CR1-EGFP knock-in mice were obtained from The Jackson Laboratory (Bar Harbor, ME, USA). The PGRP-S-DsRed mice, where DsRed expression is driven by the PGRP-S promoter (Wang et al., 2011), were bred onto the C57Bl/6 background. All mice were bred in the University of California, Riverside vivarium under specific pathogen-free conditions and were handled in accordance with Institutional Animal Care and Use Committee and National Institutes of Health guidelines.

### Implant Fabrication

The implant used in this study was shaped from a high-consistency medical-grade translucent silicone elastomer (MED-4780; NuSil, Carpinteria, CA, USA), approved for human implantation for a period of greater than 29 days. A 2 mm thick section of MED-4780 was embedded in optimal cutting temperature compound (OCT; Thermo Fischer Scientific, Waltham, MA, USA) and cut at 250 μm using a cryostat microtome, resulting in a 2 × 0.25 mm slice. The pieces were further hand-cut to have final dimensions of 3 × 1.0 × 0.25 mm and sterilized by autoclaving.

### Ear Pinna Implant Infection Model

Mice were anesthetized using ketamine-xylazine (100 mg/kg-5 mg/kg, IP; MilliporeSigma, St. Louis, MO, USA) and placed on a 36°C heat mat for the duration of the surgery. The left ear was affixed to a petri dish using double-sided adhesive tape and the dorsal side hair was removed using hair removal lotion. The ear was then sanitized with povidone-iodide or 70% ethanol and a small incision was made through the dorsal cutaneous layer with a scalpel. A pocket was then formed by gently separating the dermal layers of the ear pinna using a non-serrated specimen forceps. Next, 10^3^ CFUs of *S. aureus* in 2 μL PBS was inoculated directly into the ear pocket. In some cases, the surgery and implant were kept sterile to monitor aseptic conditions. After insertion of the implant, the incision was closed using VetBond tissue adhesive (3M, Saint Paul, MN, USA).

### Multiphoton Microscopy (MPM)

To assess both biofilm formation and innate immune cell activity, at least 3 sites directly on and adjacent to the biofilm/implant were imaged in each mouse. Time-lapse Z-stack images were acquired every 1–2 min over a 20–40 min span with 1 μM slices (times varied due to section thickness) using a 40× water immersion lens. MPM was carried out at the UC Riverside Center for Intravital Imaging, equipped with a Nikon A1R Multiphoton Plus (MP+; Nikon, Tokyo, Japan) microscope, including an auto-aligned tunable (700–1,080 nm) infrared laser (Coherent, Santa Clara, CA, USA), resonant and galvano scanners, 3 Gallium-arsenide-phosphide (GaAsP) non-descanned detectors (NDD), and 1 high sensitivity NDD for IR detection. The Nikon A1R MP+ is housed in a procedure room within the specific pathogen-free rodent vivarium and was fitted for BSL-2 usage. Still images and movies were collected, analyzed, and prepared for publication using Nikon NIS-Elements software.

### Cell Tracking and Quantification

Neutrophil migration patterns were quantified using Volocity software (Quorum Technologies, Guelph, Ontario, Canada). Automated tracking algorithms to follow cells was confirmed using manual tracking by marking each individual object at each timepoint. The measurements of each neutrophil at each timepoint was taken relative to the centroid of the region of interest and were collectively gathered to form one measurement. Several parameters to define cell behavior were assessed, including velocity, displacement, and meandering index. Velocity represents the average speed of the neutrophil over the track. Displacement represents the average straight line distance from the first timepoint centroid to the last. Meandering index measures deviation from a straight line, with values from 0 to 1.

### Post-infection Analyses

After 9 days, animals were sacrificed, and the infected ear was collected for bacterial burden enumeration associated with the implant and surrounding soft tissue. Briefly, the ear was removed, rinsed with 70% ethanol, and the excised implant placed in 100 μL PBS for sonication to dissociate bacteria from the implant surface. The ear was then weighed, cut into smaller pieces, then dissociated in 500 μL of PBS using the blunt end of a 3 mL syringe plunger. Tissue and implant bacterial titers were quantified on TSA and expressed as CFU per mL for implants and CFU per gram for tissue.

### Statistical Analyses

Significant differences between experimental groups were determined as described in the respective figure legends. GraphPad Prism 8 (GraphPad, San Diego, CA, USA) was used for all statistical analysis calculations, and a *P* < 0.05 was considered statistically significant. Biofilm images are representative from 8 independent experiments. Neutrophil tracking data are from two independent experiments with statistical analyses performed using an unpaired, two-tailed *t*-test.

## Results

**Figure 1:**
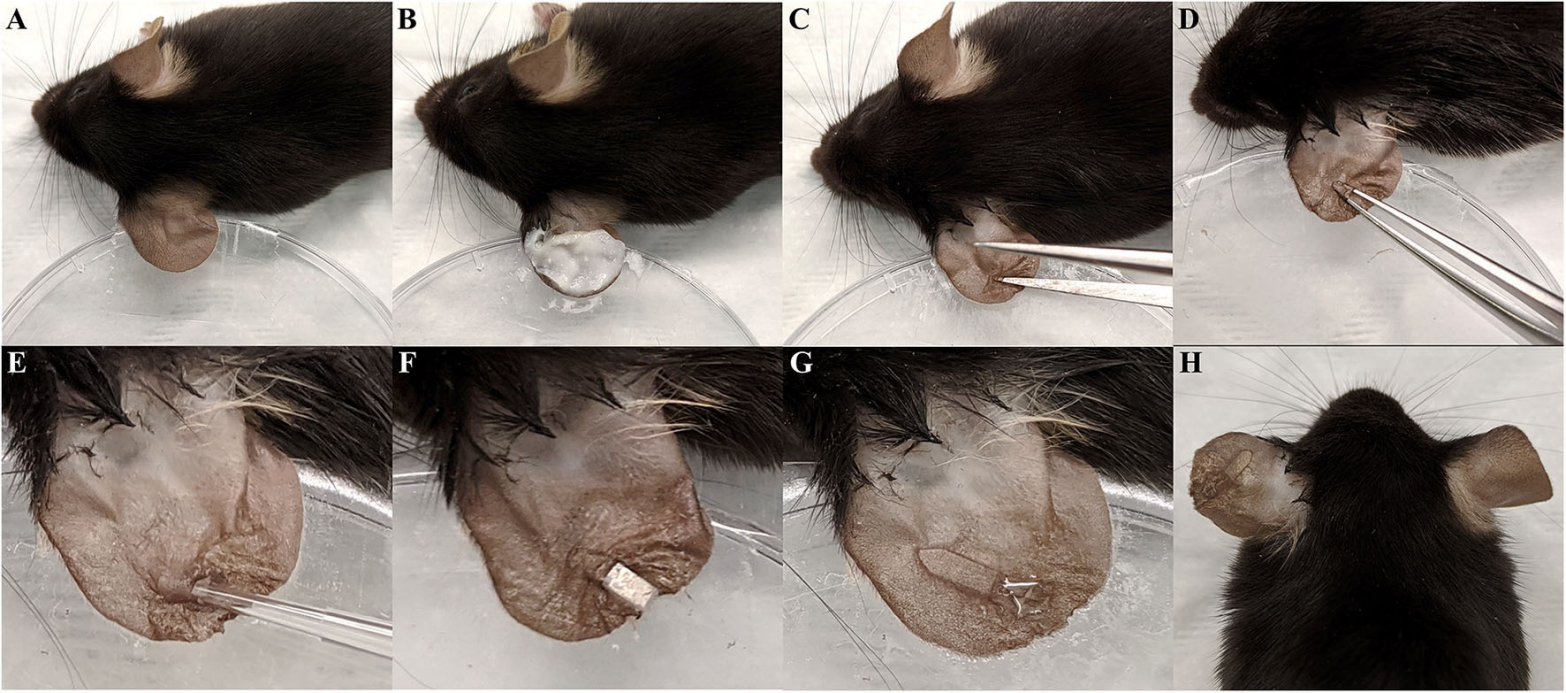
Interdermal implant -associated biofilm infection model surgery. **(A)** The left ear pinna of an anesthetized mouse was affixed to a petri dished using double-sided tape. **(B)** Dorsal side hair was removed from the ear using hair removal cream. **(C,D)** A small incision was made in the dorsal side dermis and widened into a pocket using forceps. **(E)** 10^3^ CFU of *S. aureus* in 2 μL PBS was then inoculated into the pinna pocket. **(F)** The 0.25 mm thin slice of silicone elastomer was slid into the pocket. **(G)** The incision was closed with tissue adhesive. **(H)** Final appearance of the ear following surgery and infection.

The mouse ear pinna is a proven site for assessing immune responses and infection progression (Li et al., 2012; Abtin et al., 2014; Forestier et al., 2017). Thus, we sought to establish an ear pinna interdermal implant model of *S. aureus* biofilm infection. Briefly, for the implant surgery and infection, the left ear of an anesthetized mouse was affixed to a petri dish and the dorsal side hair removed (Figures 1A,B). The ear was then sanitized, a small incision made through the outer cutaneous layer, and a small pocket formed by gently separating the dermal layers of the ear pinna (Figures 1C,D). Next, 10^3^ CFU of *S. aureus* LAC-13C harboring pCM29 was pipetted directly into the interdermal pocket (Figure 1E). Finally, a section of translucent medical grade silicone was then inserted into the ear pinna pocket and the incision closed using tissue adhesive (Figures 1F–H).

**Figure 2:**
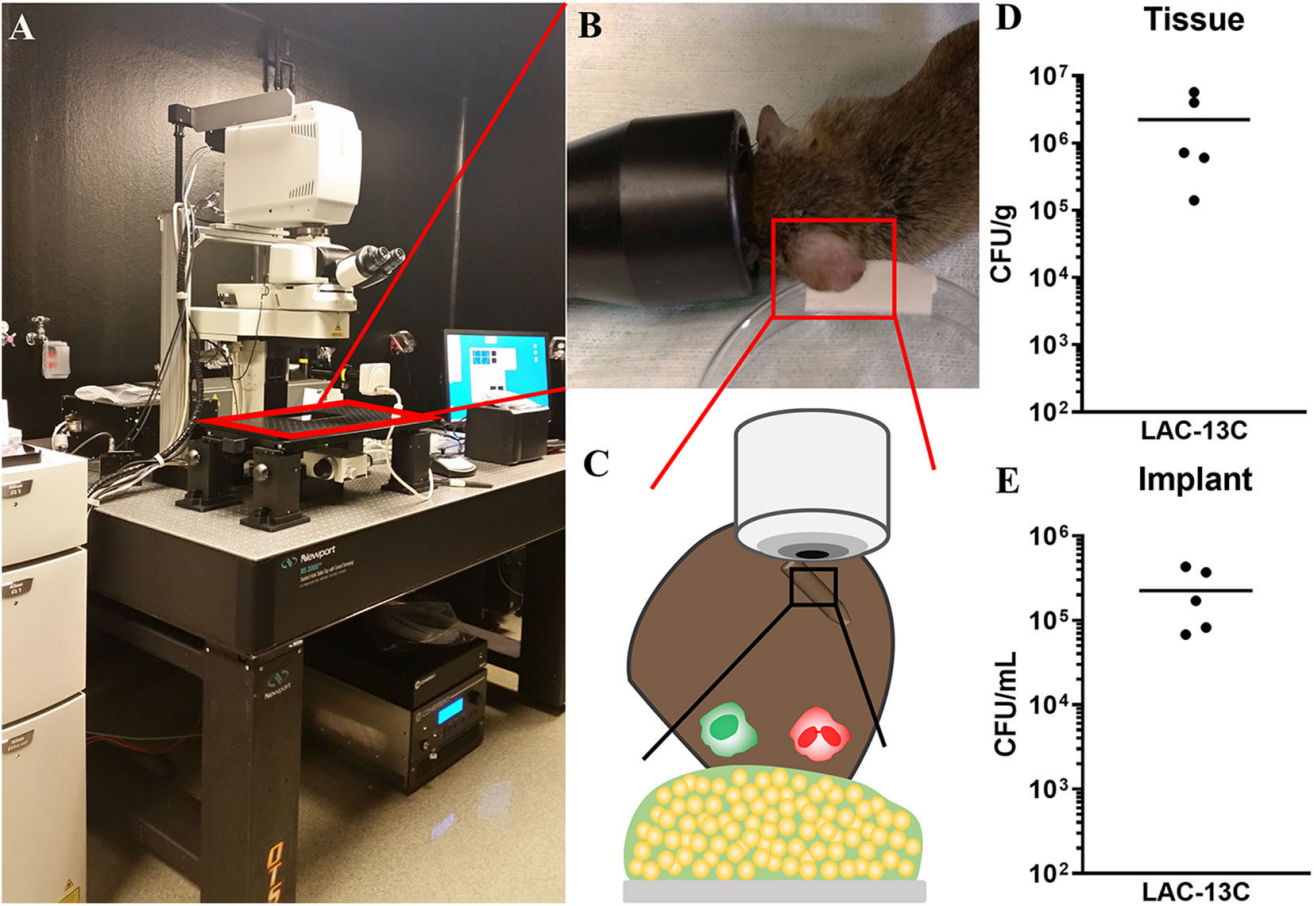
Multiphoton microscopy illustration and post-infection analysis of implant-associated *S. aureus* biofilm infection. **(A–C)** MPM arrangement with a cartoon illustration of biofilm infection visualization. **(D,E)** Day 9 post-infection analysis of bacterial burdens associated with surrounding ear tissue **(D)** and the silicone implant **(E)**. Data are from two independent experiments with the horizontal line representing the mean.

Murine device-associated *S. aureus* biofilm infections typically reach maximum bacterial burdens 3 days post-infection, with *bona fide* biofilms formed by day 7 (Thurlow et al., 2011; Heim et al., 2015; Yamada et al., 2018). Thus, intravital MPM imaging took place daily starting 2 days and ending 9 days post-infection (Figures 2A–C). Importantly, this model and time frame permits the establishment of a robust infection associated with both the implant and surrounding tissue. As shown in Figures 2D,E, tissue and implant-associated bacterial burdens after 9 days were comparable to those observed in other *S. aureus* biofilm infection models (Yamada et al., 2018; Gries et al., 2020).

**Figure 3:**
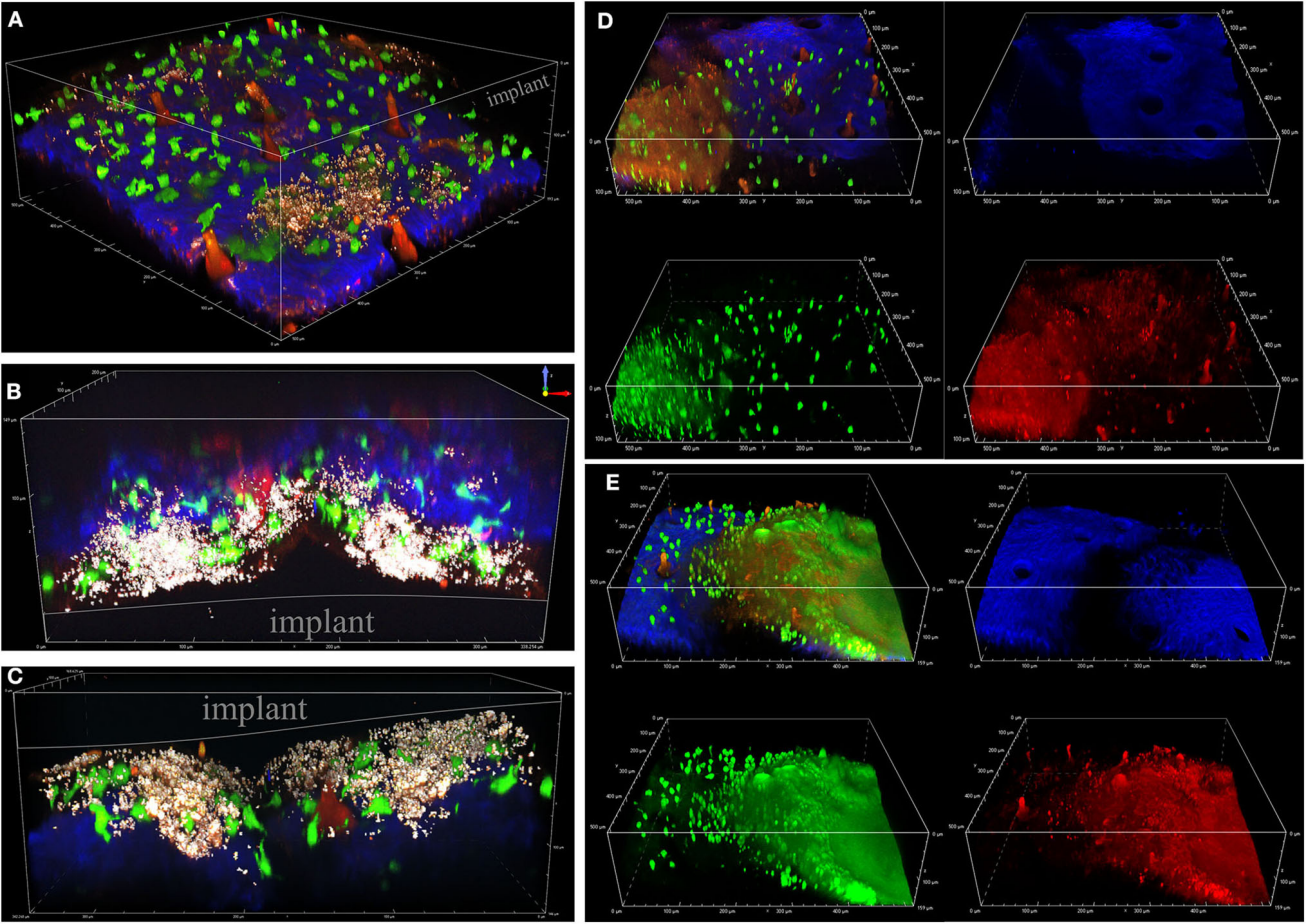
Visualizing *S. aureus* biofilm infection and immune cell interactions. Intravital MPM of a CX3CR1-EGFP reporter mouse infected with *S. aureus*, shown in bright white. EGFP^+^ macrophages/dendritic cells are shown in green, and second-harmonic generated connective tissue (blue) and vasculature (red). Bacterial cells are depicted in red-white as superfolder-GFP expression is detectable in all channels. Representative images depict biofilms at 3 **(A)**, 5 **(B,C)**, 7 **(D)**, and 9 **(E)** days post-infection, taken from 8 independent experiments.

To visualize *S. aureus* biofilm development and associated innate immune activity *in vivo*, we utilized a CX3CR1-EGFP transgenic mouse where EGFP is expressed in monocytes and dendritic cells (Jung et al., 2000). Representative images of *S. aureus* biofilm development over 9 days post-infection are shown in Figure 3. *S. aureus* cells are shown in red-white as the bright superfolder GFP emitted fluorescence in both the FITC and TRITC channels. The early stages of infection (days 3 and 5) depict a loose accumulation of individually visible *S. aureus* cells associated with the implant (Figures 3A–C). Later stages of infection (days 7 and 9) revealed a larger mass of cells encased in a hazy matrix that largely prevented the visualization of individual bacterial cells (Figures 3D,E). While the composition of this extracellular matrix remains to be determined, it is possible that this is the result of mature biofilm matrix development.

**Figure 4:**
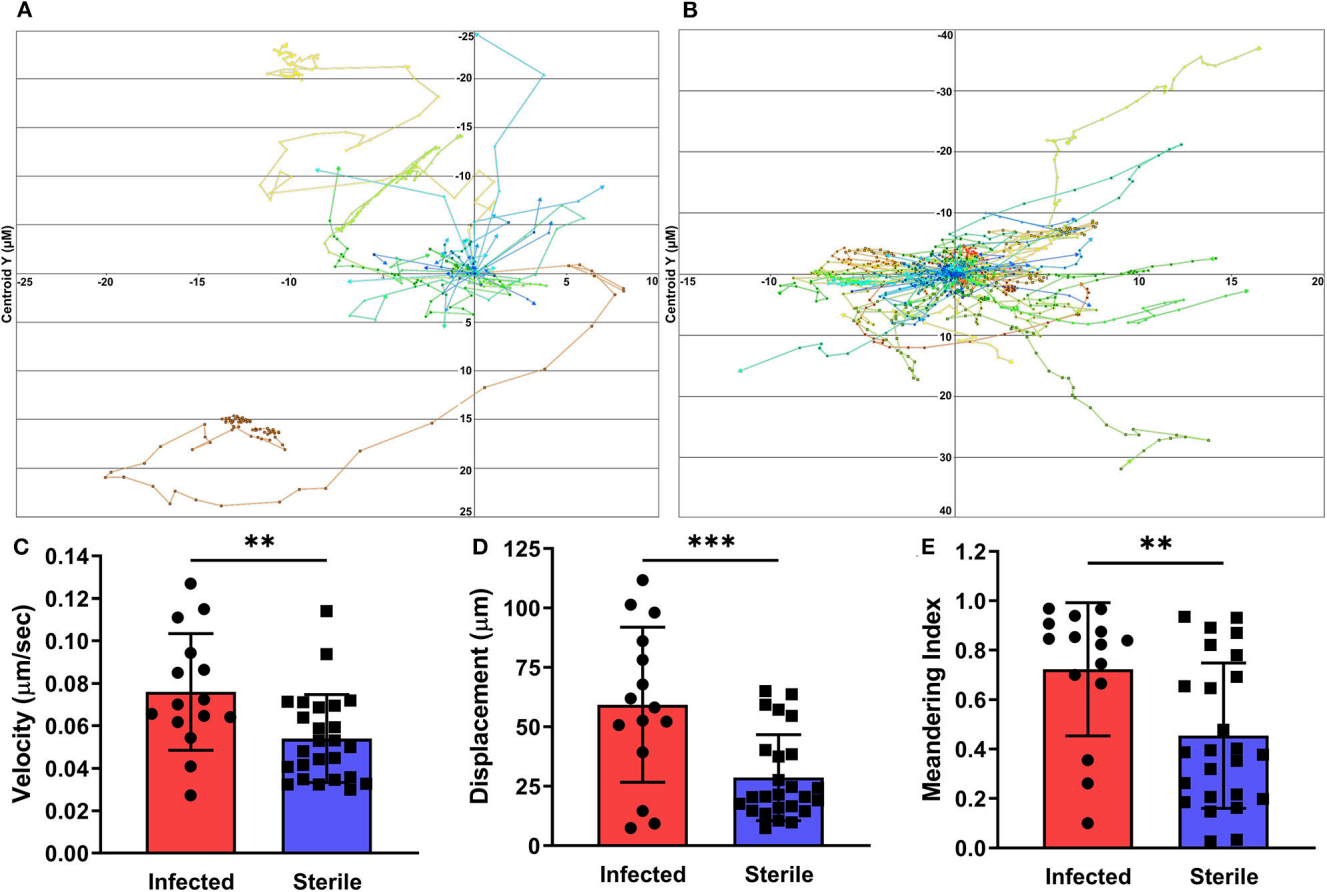
Neutrophil tracking in infected and sterile tissues. Cells immediately adjacent to *S. aureus* infected **(A)** or sterile **(B)** implants were tracked over the given experimental time span with each data point representing a separate time point. Quantification of neutrophil velocity **(C)**, displacement **(D)**, and meandering index **(E)**, from infected and sterile images, normalized to experimental duration and time between data points. Data are from two independent experiments and presented as mean ± standard deviation. Statistical analyses were performed using an unpaired, two-tailed *t*-test; **P < 0.01, ***P < 0.001.

To measure neutrophil migration patterns in response to sterile vs. biofilm infected implants, we utilized a PGRP-S-DsRed transgenic reporter mouse. The tracking paths of neutrophils associated with sterile and infected tissues on day 3 post-surgery are show in Figures 4A,B. Importantly, neutrophils near but not in direct contact with individual bacterial cells or the biofilm were assessed. Compared to neutrophils associated with a sterile implant, those near the infected implant showed significantly increased cell migration velocity and displacement (Figures 4C,D). Interestingly, these patterns were also associated with a significantly increased meandering index, or what appeared to be rapid but aimless neutrophil migration (Figure 4E). Together, these data shed light on the development of *S. aureus* biofilm during device-associated infection and the effects of the biofilm infection on neutrophil activity.

## Discussion

*Staphylococcus* sp. biofilms are the most common cause of medical device-associated infections (Arciola et al., 2018). These infections can arise in otherwise healthy individuals and often lead to chronic, reoccurring infections. Productive innate immune responses are inhibited by *S. aureus* biofilm products, including polarizing infiltrating macrophages and recruitment of myeloid-derived suppressor cells (Scherr et al., 2015; Gries and Kielian, 2017). Moreover, the three-dimensional biofilm architecture itself presents a barrier to immune cell infiltration and phagocytosis *in vitro* (Thurlow et al., 2011; Gries et al., 2020). However, relatively little is known of the host-pathogen interface during *S. aureus* biofilm-mediated infection. In this work we demonstrate the capability of multiphoton microscopy (MPM) to assess bacterial biofilm development and measure associated neutrophil migratory activity. To date, this is the first report using MPM to assess device-associated bacterial biofilm-mediated infection and concomitant innate immune responses.

The mouse ear pinna presents an ideal tissue to examine biofilm development and is a proven site to analyze innate immune activity using MPM (Li et al., 2012). Relative to the mouse body, the ear is sparse in auto-fluorescent hair coverage which can be easily removed prior to surgery or imaging. Furthermore, the implant/infection surgery causes minimal tissue damage and requires few specialized tools. Finally, MPM of the ear pinna is non-invasive and permits extended time-lapse imaging over multiple days using the same animal. In our model, the CX3CR1-EGFP and PGRP-S-DsRed transgenic animals expedite the orientation and focus within the pinna tissue. The *S. aureus* superfolder GFP used in these studies is highly fluorescent and easily detectable in both the FITC and TRITC channels. While this may present some future challenges, it allows easy detection of bacterial cells in the context of second harmonics and mouse fluorescent reporter expression. Notably, we did not measure any significant loss of the GFP plasmid over the 9-day period (data not shown).

Using MPM, we sought to examine the developmental stages of *S. aureus* biofilm formation *in vivo* and simultaneously examine innate immune cell responses. This is a necessary step as multiple *in vitro* models of biofilm/immune cell co-culture have been employed, but their accuracy to biofilm infection has not been well-established. While many device-associated infections are multispecies, we limited this study to assess *S. aureus* biofilms and their impact on innate immune cell function. We showed that *S. aureus* device-associated infections follow a developmental progression and time frame analogous to that observed *in vitro* using minimal media such as RPMI (Thurlow et al., 2011; Gries et al., 2016). Initially, single *S. aureus* cells are easily observable and closely associated with the implant. By day 5, the number of visible bacterial cells was markedly increased. It remains unclear why maximum bacterial burdens are typically seen by day 3 post-infection, yet a considerable difference exists in the number of bacteria observed between days 3 and 5. A possible explanation is that bacterial viability was substantially decreased between these time points, however further examination of viability would be required to delineate this observation.

Mature *S. aureus* biofilms are characterized as having an extensive extracellular matrix composed of various proteins, sugars, and extracellular DNA (Foulston et al., 2014; Lister and Horswill, 2014). It was therefore not surprising to find that after 7 days, an extensive, hazy covering of the bacterial cells appeared. The matrix would also likely consist of a significant portion of GFP molecules, potentially providing its robust fluorescent appearance. An interesting observation throughout the time course is the overall lack of phagocytosis by monocytes, dendritic cells, and neutrophils. While this is in agreement with *in vitro* findings (Thurlow et al., 2011; Gries et al., 2020), a paucity of phagocytosis was observed beginning on day 3 post-infection, before the robust biofilm structure was formed. Further investigation will be required to assess the role of *S. aureus* biofilm molecules known to affect macrophage and neutrophil phagocytic function (Scherr et al., 2015; Gries et al., 2016; Bhattacharya et al., 2018).

Neutrophils and macrophages are essential cellular components of the innate immune response to bacterial biofilm infection. Their presence also markedly alters *S. aureus* biofilm gene expression *in vitro* (Scherr et al., 2013). Neutrophils are the first line of cellular immune defense against invading bacterial pathogens; their coordinated recruitment and activity are essential to preventing and eliminating infection. Compared to macrophages, neutrophils have shown a much higher propensity for biofilm invasion and phagocytosis *in vitro* (Gunther et al., 2009; Thurlow et al., 2011; Ghimire et al., 2019). We observed significant differences in the cell migration behaviors of neutrophils in response to infected versus sterile implants. Despite neutrophils appearing to respond to biofilm infection by increasing migration velocity and displacement, their movements were less directed toward the implant/infection than neutrophils in sterile tissues. These observation are consistent with a recent report that a delay in neutrophil recruitment to the implant surface may permit *S. aureus* time to grow and form biofilm (Ghimire et al., 2019). We anticipate that neutrophil deviation is a result of *S. aureus* biofilm products interfering with neutrophil cytokine/chemokine signaling and the production of multiple leukocyte inhibitors and toxins (Rooijakkers et al., 2006). The mechanism(s) responsible for neutrophil redirection away from *S. aureus* biofilm are subject of future investigations. Together, these observations further establish the ineffective nature of neutrophil responses to *S. aureus* biofilm-mediated infection.

## Data Availability Statement

The raw data supporting the conclusions of this article will be made available by the authors, without undue reservation.

## Ethics Statement

The animal study was reviewed and approved by Institutional Animal Care and Use Committee.

## Author Contributions

CG and DL conceived and designed the experiments. CG, ZR, and JC performed the experiments. CG, ZR, JC, and DL analyzed data. CG wrote the paper. All authors contributed to the article and approved the submitted version.

## Conflict of Interest

The authors declare that the research was conducted in the absence of any commercial or financial relationships that could be construed as a potential conflict of interest.
